# Surgical Management of Pachydermodactyly (PDD) via Midaxial Incision: A Case Report

**DOI:** 10.7759/cureus.25802

**Published:** 2022-06-09

**Authors:** Ayumi Sakai, Makoto Omori, Misato Ueda

**Affiliations:** 1 Plastic Reconstructive Surgery, Kobe University Graduate School of Medicine, Kobe, JPN; 2 Plastic Surgery, Yodogawa Christian Hospital, Osaka, JPN

**Keywords:** case report, surgery, midaxial incision, proximal interphalangeal joints, pachydermodactyly

## Abstract

Pachydermodactyly (PDD), meaning “thick skin finger” in Greek, is a rare, noninflammatory, benign, superficial fibromatosis. We report the case of PDD in a 15-year-old boy who visited our clinic because of asymptomatic swelling of the proximal interphalangeal (PIP) joints on the third finger of both left and right hands. Physical examination revealed thickening of the skin in the radial and ulnar aspects of the PIP joints of his third finger of both hands without functional limitation or neurological symptoms. He had a habit of biting his swelling fingers, and he belonged to a basketball club at his junior high school. He had no medical history. Plain radiographs and magnetic resonance imaging of both hands showed only soft tissue thickening outside of the radial and ulnar collateral ligament of the bilateral third PIP joint. The lesions were suggestive of PDD. Surgical resection was performed via a midaxial incision and a Z-plasty to confirm the diagnosis and improve the aesthetic appearance of his hands. Histopathological examination of the lesions was compatible with PDD. After surgery on the left hand, the patient underwent the same surgery on the right hand. No recurrence or complications were observed at the one-year follow-up after surgical intervention. Thus, surgery for PDD via a midaxial incision may be a good treatment option for patients who wish to rectify the appearance of their digital deformity.

## Introduction

Pachydermodactyly (PDD), which refers to “thick skin finger” in Greek, is a rare, noninflammatory, benign, superficial fibromatosis first reported in 1973 by Bazex [1]. The condition manifests in the radial and ulnar aspects of the proximal interphalangeal (PIP) joints of both left and right hands without damage to joint structures or the bone. Presently, there is no proven effective treatment for PDD, and nonsurgical treatment is often recommended because PDD is a benign tumor. Additionally, some patients prefer surgical intervention for aesthetic reasons. However, there have been few reports on surgery for PDD [2,3]. Herein, we present a case of successful surgical excision of PDD in a 15-year-old boy.

## Case presentation

**Figure 1:**
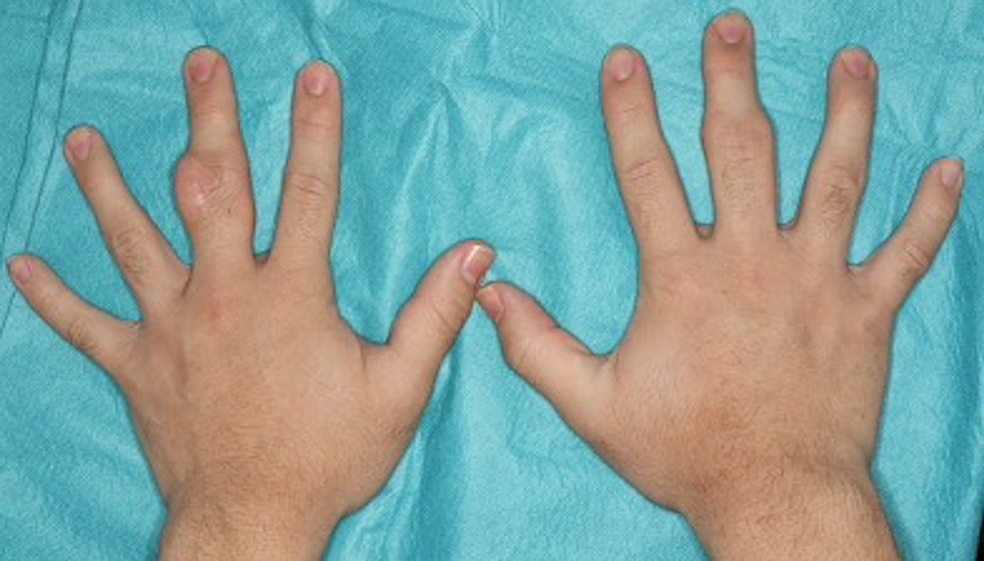
Clinical findings Soft tissue was swelling around the proximal interphalangeal (PIP) joints of the third finger on both hands.

A 15-year-old boy was referred to our plastic reconstructive surgery department due to asymptomatic swelling of the PIP joints on the third finger of both hands following several years of progressive development. He began biting the swelling lesions, especially those on the left hand. He was part of a basketball club at his junior high school. He had no medical history of obsessive-compulsive disorders or psychiatric conditions and no family history of connective tissue disease. Physical examination revealed thickening of the skin in the radial and ulnar aspects of the PIP joints of his third finger on both hands without functional limitation or neurological symptoms (Figure 1). 

**Figure 2:**
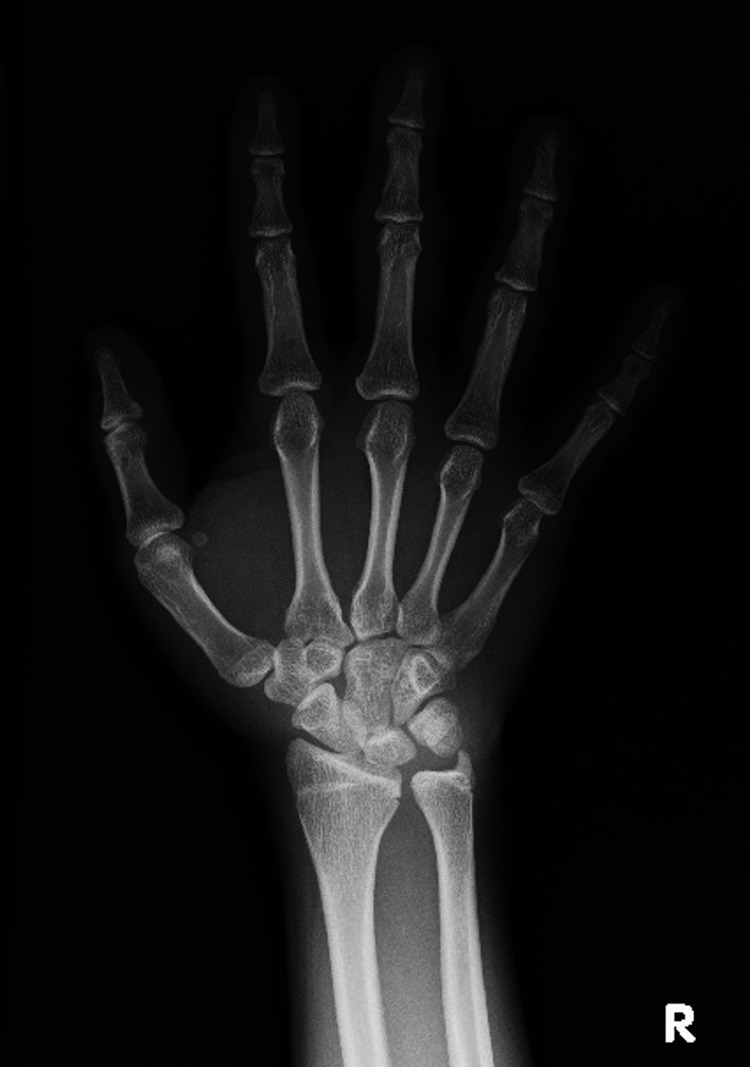
Plain radiograph Plain radiographs of the hands showing no bone destruction or joint space narrowing.

**Figure 3:**
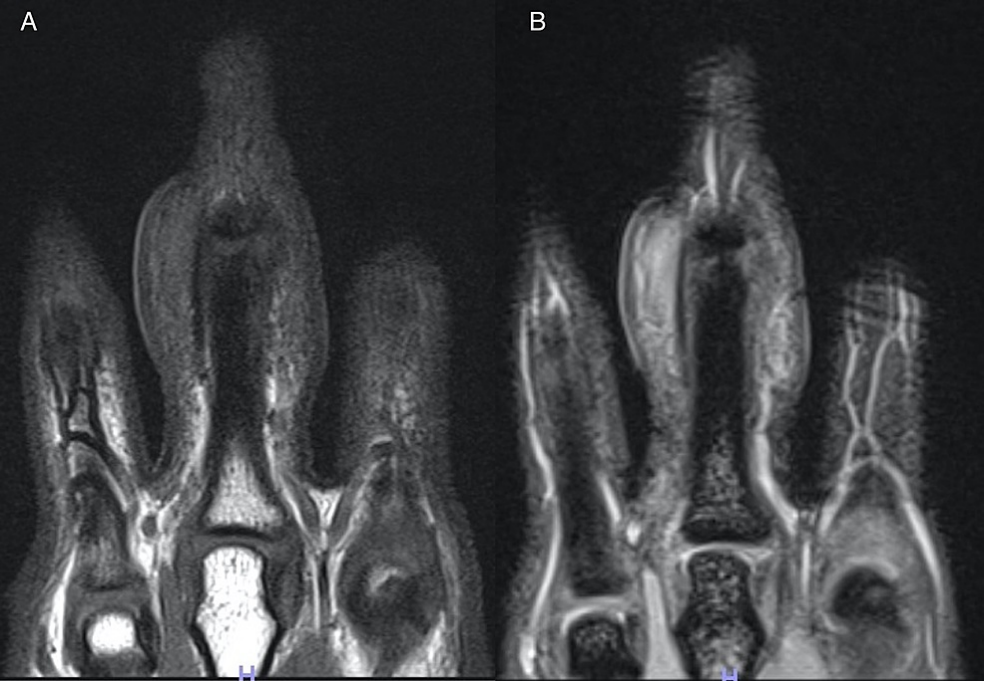
Magnetic resonance imaging Magnetic resonance imaging revealed soft tissue thickening outside of the radial and ulnar collateral ligament of the bilateral third proximal interphalangeal (PIP) joint. The bone cortex, ligaments, and joint space are intact. A) On a T1-weighted image, the lesions were hypointense. B) On a STIR T2-weighted image, the lesions were not as hyperintense.

Soft tissue swelling was more prominent on the left hand. Plain radiographs of the hands showed no bone destruction or joint space narrowing (Figure 2). Magnetic resonance imaging of the hands revealed soft tissue thickening outside of the radial and ulnar collateral ligament of the PIP joints of the third finger on both hands. Bone cortex, ligaments and joint space were intact. On T1-weighted image, the lesions appeared hypointense, whereas on STIR T2-weighted image, they did not appear as hyperintense as water (Figure 3A, B). 

**Figure 4:**
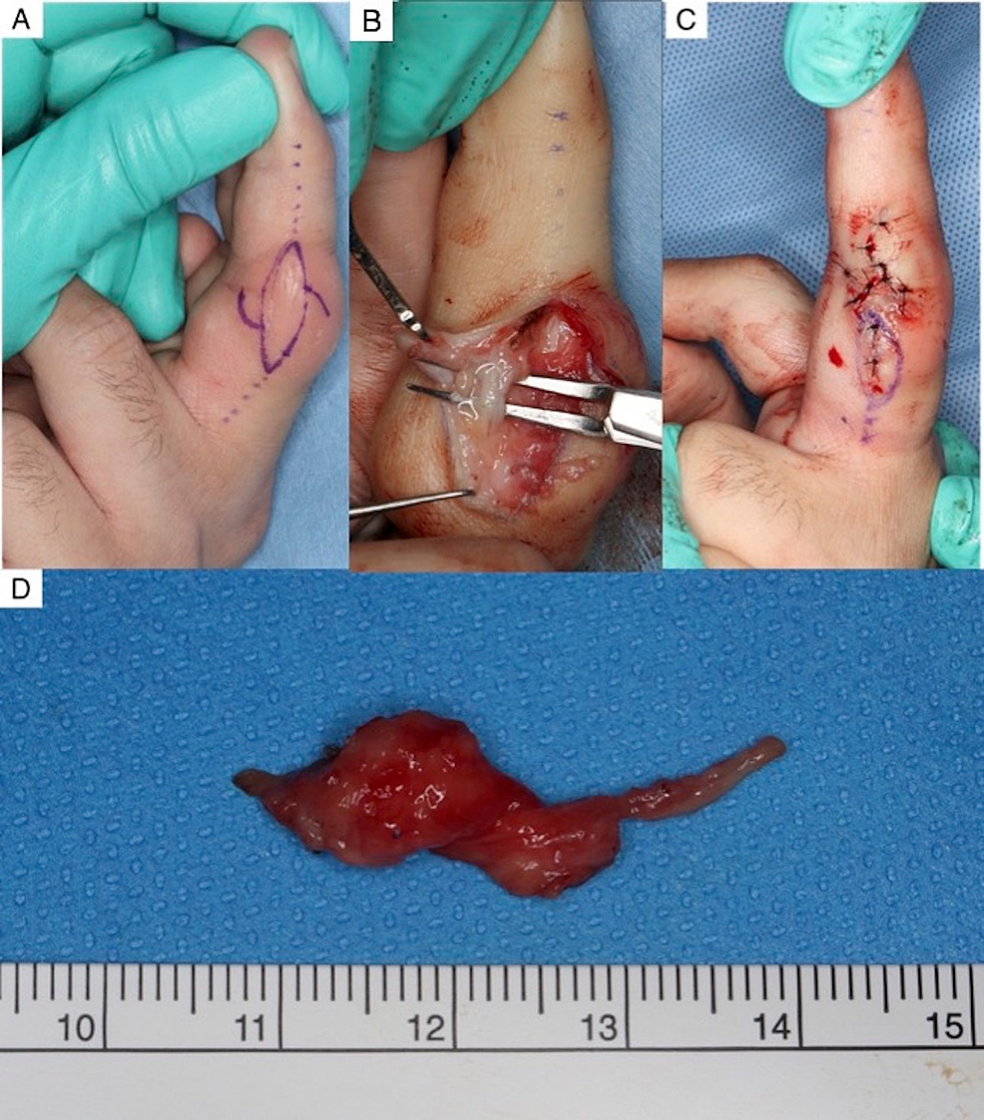
Operation A) The design of midaxial incision with a Z-plasty. B) The lesion was located outside of the radial and ulnar collateral ligament of the proximal interphalangeal (PIP) joint of the third finger. The neurovascular bundles were located at the palmar and inner sides of the lesion. C) The surgical site was closed with 5-0 nylon. D) Gross examination showed that the lesion was composed of soft tissue thickening.

The lesions were suggestive of PDD. The patient requested surgical excision to confirm the diagnosis and improve the esthetic appearance of his hands. Under digital block, the lesions on his left hand were dissected on the radial and ulnar collateral ligament and removed as a mass via a midaxial incision to preserve the neurovascular bundles. The neurovascular bundles were located at the palmar and inner sides of the lesion. A cord which was suspected to be the dorsal branch of the digital nerve running from the palmar to the dorsal side at deeper layer of the lesion, was preserved. We trimmed the excess skin and closed the incision by creating a Z-plasty at the radial and ulnar aspects of the PIP joints (Figure 4A-D).　

**Figure 5:**
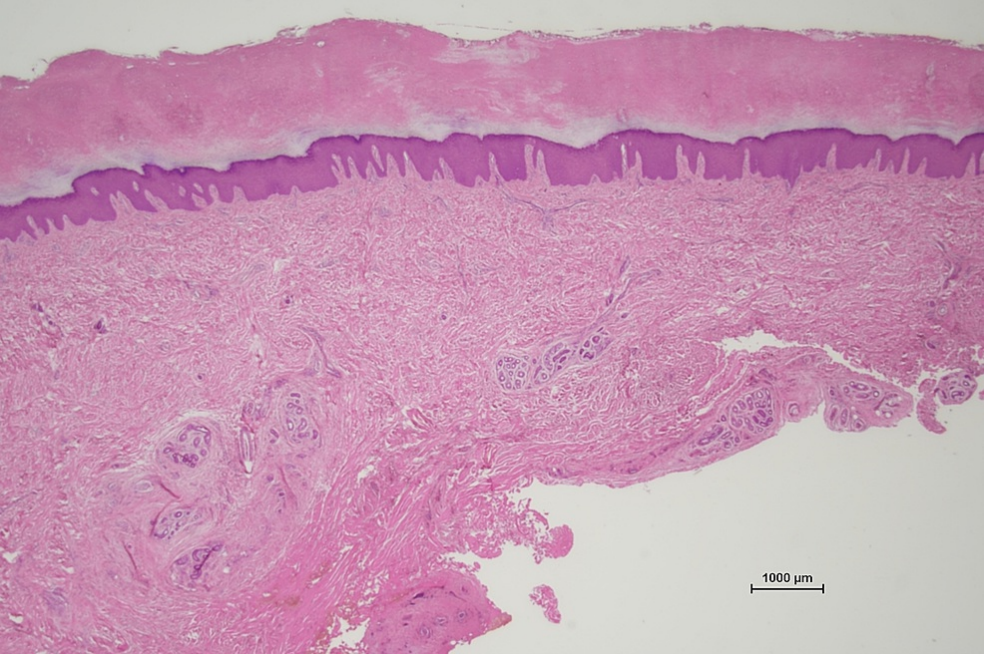
Histopathological tests Histopathological tests revealed hyperkeratosis and thickening of the dermis.

Histopathologically, the lesions were characterized by orthokeratotic hyperkeratosis, acanthosis, and thickening of the dermis owing to an increased number of fibroblasts and collagen with little infiltration of lymphocytes. These results are compatible with a diagnosis of PDD (Figure 5).

**Figure 6:**
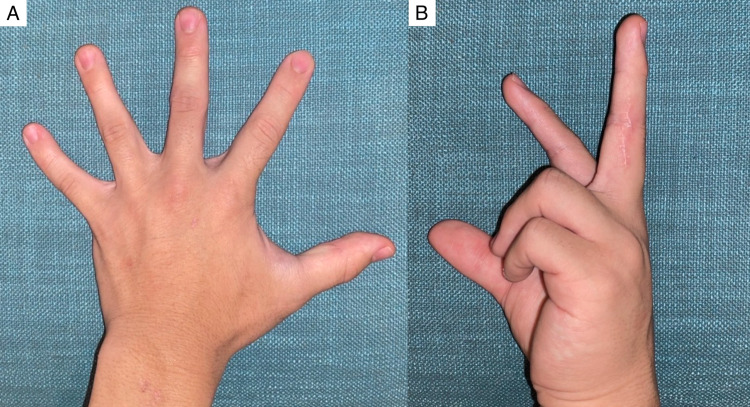
Clinical manifestations after surgery A) The contour of the digits is significantly improved. The scar is imperceptible on the dorsal and ventral surfaces of the hand. B) The patient has no movement limitations.

After surgery on the left hand, the patient underwent the same surgery on the right hand. No recurrence was observed at the one-year follow-up after surgical intervention. The patient was satisfied with the contour of his fingers and had no sensory paralysis in the dorsal area of the finger or movement limitations (Figure 6A, B).

## Discussion

The etiology of PDD is unknown, but minimal repetitive trauma due to work or habits may be related to its onset. Some authors have reported that PDD is related to neuropsychiatric disorders [4]. The common presentation is swelling around the PIP joints of the third finger, sometimes in the distal interphalangeal or metacarpophalangeal joints of all fingers, without damage to the joint structures, coupled with no morning stiffness, painless motion, and no tenderness to palpation. Often, laboratory test results are normal, and radiographs/magnetic resonance imaging show soft tissue swelling only [5]. Histopathologic features are characterized by orthokeratotic or parakeratotic hyperkeratosis, acanthosis, and thickening of the dermis owing to an increased number of fibroblasts and collagen types III and V [4,6]. The patient’s habits, finger manipulation history, clinical features, radiographs/magnetic resonance imaging, and histologic features were typical of PDD. His habit of biting his fingers, especially those on the left hand, may have resulted in larger lesions.

**Figure 7:**
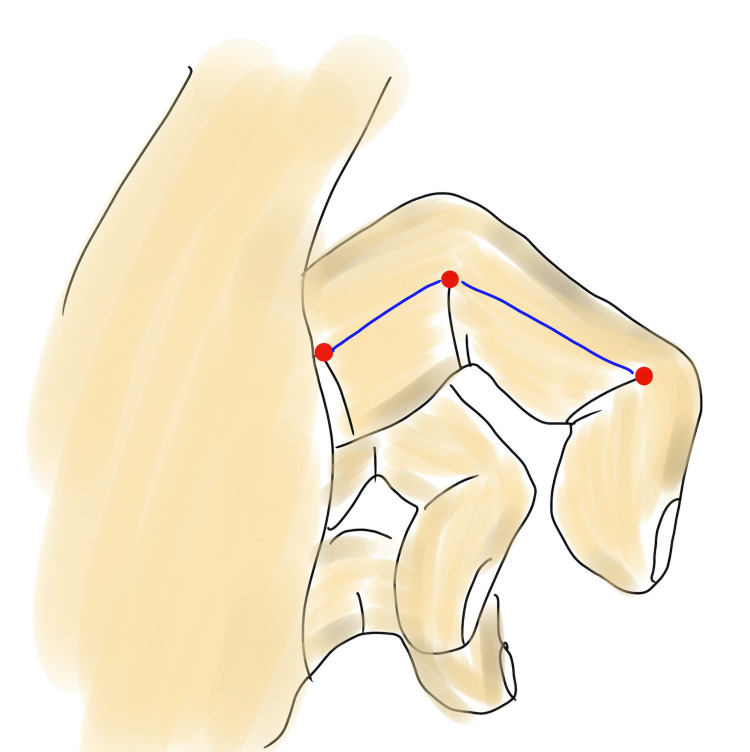
Midaxial line Midaxial line (blue line) is determined by connecting the apices of the flexion crease (red points).

To the best of our knowledge, no consensus exists regarding the treatment of PDD. Nonsurgical treatment, such as cessation of mechanical trauma to the skin, is prioritized owing to the benign nature of the tumor [4]. Another nonsurgical treatment modality involves the use of intralesional triamcinolone injection, or post-oral collagen-synthesis inhibitors such as tranilast [6]. However, these provide limited improvement in the swelling of the fingers. One case outlined surgical resection for PDD and achieved good results via the volar zig-zag approach [3]. Herein, the lesions were resected via a midaxial incision. Midaxial line is determined by connecting the apices of the flexion crease, noting the point of change between the dorsal and palmar aspects, and noting the finger in full flexion (Figure 7) [7]. 

Incision at the midaxial line is recommended for PDD as it results in no skin tension, full flexion or extension, and imperceptible scarring. The dorsal branch of the digital nerve of the third finger branches at the proximal phalanx (87.5%), metatarsophalangeal joint (10%), or palm (2.5%) [8]. Thus, close attention should be paid to this branch when using the midaxial incision approach. As PDD arises from the epidermis and dermis, it should be located within the outer layer rather than at the neurovascular bundles [4,6]. However, to the best of our knowledge, there are no reports regarding the relationship between PDD and neurovascular bundles. This may be because nonsurgical treatment is often prioritized. In the present case, neurovascular bundles were present at the palmar side of the lesion, and the dorsal branch of the digital nerve branched at the proximal phalanx, close to the deeper layer of the lesion. Therefore, both the nerves were preserved.

A Z-plasty was performed to avoid contracture. However, as a midaxial incision is originally a method of avoiding contracture, further assessment for the combined use of Z-plasty with this incision is necessary.

## Conclusions

We experienced a case that achieved a good result for digit deformity of PDD after undergoing surgery via a midaxial incision. This incision is imperceptible scarring without any complications. Thus, a midaxial incision may be a good approach for patients who wish to rectify the appearance of their digital deformity.
